# Binding mechanisms of half-sandwich Rh(III) and Ru(II) arene complexes on human serum albumin: a comparative study

**DOI:** 10.1007/s00775-019-01683-0

**Published:** 2019-07-12

**Authors:** Orsolya Dömötör, Éva A. Enyedy

**Affiliations:** Department of Inorganic and Analytical Chemistry, Interdisciplinary Excellence Centre, University of Szeged, Dóm tér 7, 6720 Szeged, Hungary

**Keywords:** Albumin, Binding affinity, Capillary electrophoresis, Fluorescence, Ultrafiltration

## Abstract

**Abstract:**

Various half-sandwich ruthenium(II) arene complexes and rhodium(III) arene complexes have been intensively investigated due to their prominent anticancer activity. The interaction of the organometallic complexes of Ru(η^6^-*p*-cymene) and Rh(η^5^-C_5_Me_5_) with human serum albumin (HSA) was studied in detail by a combination of various methods such as ultrafiltration, capillary electrophoresis, ^1^H NMR spectroscopy, fluorometry and UV–visible spectrophotometry in the presence of 100 mM chloride ions. Binding characteristics of the organometallic ions and their complexes with deferiprone, 2-picolinic acid, maltol, 6-methyl-2-picolinic acid and 2-quinaldic acid were evaluated. Kinetic aspects and reversibility of the albumin binding are also discussed. The effect of low-molecular-mass blood components on the protein binding was studied in addition to the interaction of organorhodium complexes with cell culture medium components. The organometallic ions were found to bind to HSA to a high extent via a coordination bond. Release of the bound metal ions was kinetically hindered and could not be induced by the denaturation of the protein. Binding of the Ru(η^6^-*p*-cymene) triaqua cation was much slower (ca. 24 h) compared to the rhodium congener (few min), while their complexes interacted with the protein relatively fast (1–2 h). The studied complexes were bound to HSA coordinatively. The highly stable and kinetically inert 2-picolinate Ru(η^6^-*p*-cymene) complex bound in an associative manner preserving its original entity, while lower stability complexes decomposed partly or completely upon binding to HSA. Fast, non-specific and high-affinity binding of the complexes on HSA highlights their coordinative interaction with various types of proteins possibly decreasing effective drug concentration.

**Graphic abstract:**

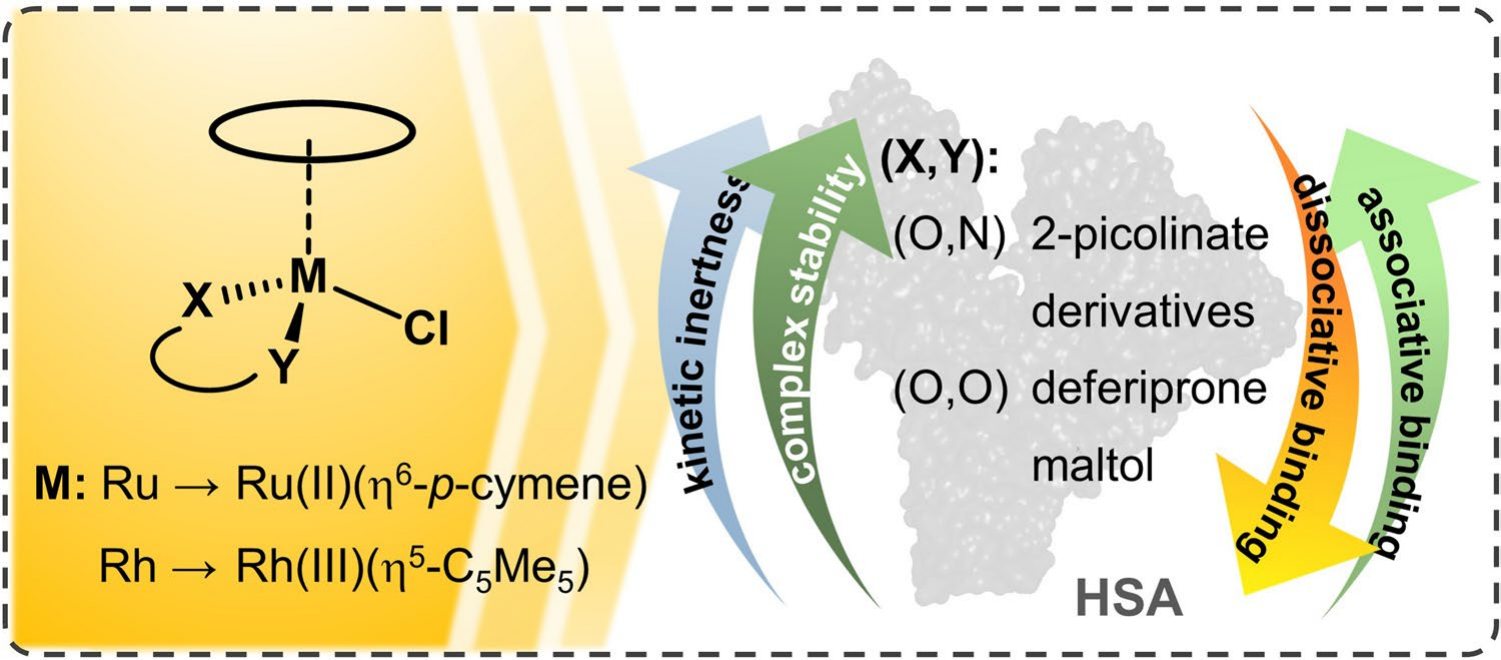

**Electronic supplementary material:**

The online version of this article (10.1007/s00775-019-01683-0) contains supplementary material, which is available to authorized users.

## Introduction

During the development of metal compounds for medicinal purposes, organometallics have been shown to exhibit promising activity in various diseases [1–5]. In particular, half-sandwich Ru(II)-arene complexes have attracted attention, since some representatives show significant anticancer activity and non-cross resistance with cisplatin. [3, 6–8]. The interest for organoruthenium compounds in cancer treatment became more pronounced as the clinically relevant Ru(III) drug candidates NKP1339 and NAMI-A were reported to exert their anticancer effect in the reduced Ru(II) forms [9]. Among Ru(II) compounds [Ru(II)(4,4′-dimethyl-2,2″-bipyridine)(2-((2′,2′’:5′’,2′’’-terthiophene)-imidazo[4,5-f] [1,10] phenanthroline))] (TLD1433) has completed a phase 1 clinical trial for treating bladder cancer with photodynamic therapy [10]. [Ru(II)(η^6^-*p*-cymene)(PTA)Cl_2_] (RAPTA-C; PTA = 1,3,5-triaza-7-phosphaadamantane) and the RAED type [Ru(II)(η^6^-biphenyl)(ethylenediamine)Cl]^+^ (RM175) are considered the lead structures for antitumor half-sandwich Ru(II)-arene compounds [1, 5, 11, 12]. Complexes of the isoelectronic congener Rh(III)(η^5^-C_5_Me_5_) showing promising in vitro anticancer activity have also been intensively investigated [13, 14]. Early reports on the mechanism of action of anticancer Ru(III) complexes suggested interaction with DNA, similar to cisplatin, which was believed to be ultimately responsible for the anticancer activity. Even in the case of Ru(II)-arene complexes, DNA nucleobases were primary suspects in their cytotoxic activity [15–18]. Although the actual mechanisms of action of these complexes are still not clarified, in recent years, more studies evidenced that DNA is not necessarily the only and/or primary target for ruthenium organometallics. Currently, the role of intra and extracellular proteins as possible targets of organoruthenium and related complexes is widely investigated [19].

Binding of these complexes to proteins is of considerable interest not only because of the exploration of feasible modes of action but explanation of side effects and pharmacokinetic behavior becomes possible as well. Amino acid side chains of proteins, similarly to DNA nucleobases, provide versatile coordinating sites for metal ions. The question is how these Ru(η^6^-arene) and Rh(η^5^-arenyl) complexes can interact with protein donor groups. Thus, our research interests are in the area of the following: (i) what kind of adducts can be formed, (ii) which are the preferred sites and, (iii) what are the time frame and the extent of these interactions. If these complexes are administered intravenously, potential first binding partners in vivo are blood constituents such as the non-specific transport protein albumin. In case of oral administration after the absorption from the gastrointestinal tract, the drug also reaches the systemic circulation. In both cases, binding to transport proteins, e.g., to human serum albumin (HSA) has a profound effect on the distribution, metabolism and excretion of a compound. This interaction can be advantageous due to the enhanced permeability and retention effect in solid tumor tissues resulting in the accumulation of protein-bound drugs close to the cancer cells [20]. On the other hand, an irreversible protein binding can decrease effective drug concentration and may be responsible for adverse effects as well [21, 22]. Notably, the cell culture media used for the in vitro cytotoxic measurements also contain albumin and other serum proteins, thus interaction with these proteins in the medium can also affect the original integrity of the complex.

HSA binding of anticancer metallodrugs is a widely investigated area and numerous papers were published for, e.g., Pt(II) [23–26], Ru(III) [27–31] and Ga(III) [32–34] complexes. In vitro studies on direct albumin binding of half-sandwich organometallic complexes are also reported [35, 36], however, in vivo results are rarely published [37]. Klose et al. have investigated recently the distribution of two half-sandwich *p*-cymene complexes ([Ru(η^6^-*p*-cymene)(plecstatin)Cl] and [Os(η^6^-*p*-cymene)(plecstatin)Cl]) in mouse blood serum by size-exclusion chromatography–inductively coupled plasma–mass spectrometry (SEC–ICP–MS). Free drug was not observed in any of the samples indicating rapid protein binding of the metallodrugs. A larger portion of these drugs (ca. 75%) was found in the albumin/transferrin fraction underlying the importance of interactions towards HSA as well [37].

**Chart 1:**
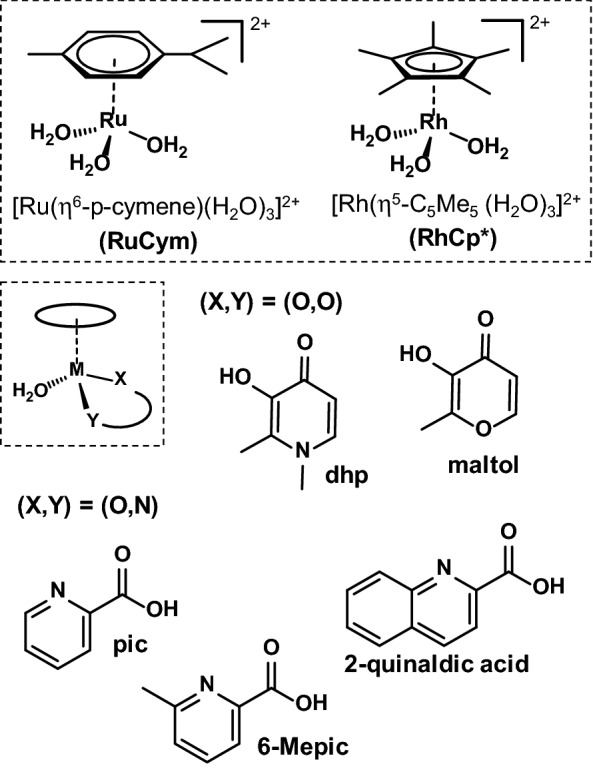
Chemical structures and abbreviations of the organometallic triaqua cations of Ru(η^6^-*p*-cymene) (RuCym) and Rh(η^5^-C_5_Me_5_) (RhCp*), general formula of the studied complexes and structures of the complex forming bidentate ligands in their neutral forms: 1,2-dimethyl-3-hydroxy-pyridin-4(1H)-one (deferiprone, dhp), 3-hydroxy-2-methyl-pyran-4(1H)-one (maltol), 2-picolinic acid (pic), quinoline-2-carboxylic acid (2-quinaldic acid) and 6-methyl-2-picolinic acid (6-Mepic)

Sadler et al. reported the coordination of the rather accessible surface imidazole nitrogen donors (histidines) of HSA to the Ru(II) center in the case of RM175 [38], while adduct formation with cytochrome c was thought to involve the N-terminus or carboxylic acid side chains on the protein [39]. Moreover, several single crystal X-ray structures confirm coordinative binding of various types of Ru(η^6^-*p*-cymene) and Rh(η^5^-C_5_Me_5_) complexes towards proteins like lysozyme, apo-ferritin or histone proteins [40–48], which are not believed to be all the primary targets for these organometallic compounds but may provide insight on the types of non-specific adducts formed with proteins. The albumin binding of Rh(η^5^-C_5_Me_5_) and its complexes formed with 1,2-dimethyl-3-hydroxy-pyridin-4(1H)-one (deferiprone, dhp), ethylenediamine and 2,2′-bipyridine (bpy) was formerly investigated in our research group. Our results suggested that the interaction with HSA can proceed in both dissociative (binding of the organometallic fragment only) and non-dissociative manner depending most probably on the thermodynamic stability of the metal complexes [49]. HSA is a key protein in blood transport and may serve as a universal protein model as well. Herein we provide a comprehensive picture on the interactions of various Rh(η^5^-C_5_Me_5_), Ru(η^6^-*p*-cymene) complexes with HSA. Studied compounds are the organometallic cations [Rh(η^5^-C_5_Me_5_)(H_2_O)_3_]^2+^ and [Ru(η^6^-*p*-cymene)(H_2_O)_3_]^2+^ themselves and their complexes formed with bidentate ligands: 3-hydroxy-2-methyl-pyran-4(1H)-one (maltol), dhp, 2-picolinic acid (pic), 6-methyl-2-picolinic acid (6-Mepic) and quinoline-2-carboxylic acid (2-quinaldic acid) (Chart 1). Our goal is to provide a comprehensive overview of the binding strength, location, and kinetics and the binding mode and its reversibility. The effect of low-molecular-mass components on the protein binding and the stability of selected complexes in a minimum essential cell culture medium were tested as well. Efficiency and possible shortages of techniques applied here, namely ^1^H NMR-, UV–visible and fluorescence spectroscopies; ultrafiltration–UV–visible and capillary zone electrophoresis, are critically discussed as well.

## Experimental

### Chemicals

[Rh(η^5^-C_5_Me_5_)(µ-Cl)Cl]_2_, [Ru(η^6^-*p*-cymene)(µ-Cl)Cl]_2_, maltol, dhp, pic, 6-Mepic, 2-quinaldic acid, bpy warfarin (WF), dansylglycine (DG), sodium dodecyl sulfate (SDS), citric acid, sodium lactate, l-amino acids: histidine (His), methionine (Met), cysteine (Cys), serine (Ser), and inorganic salts KCl, KH_2_PO_4_, NaH_2_PO_4_, Na_2_HPO_4_ were products of Sigma-Aldrich or Reanal in puriss quality. HSA (A8763, essentially globulin free) and Roswell Park Memorial Institute (RPMI) 1640 cell culture medium was purchased from Sigma-Aldrich.

### Stock solutions and sample preparation

Milli-Q water was used for preparation of stock solutions and samples. Exact concentrations of stock solutions of the ligand and the organometallic cations were determined by pH-potentiometric titrations according to procedure published in our former work [50]. Organometallic complexes were obtained by mixing the corresponding ligand and metal precursor ([Rh(η^5^-C_5_Me_5_)(µ-Cl)Cl]_2_, [Ru(η^6^-*p*-cymene)(µ-Cl)Cl]_2_) in 1:0.5 molar ratio in water; solutions were equilibrated for 24 h. HSA stock solutions were prepared in modified phosphate buffered saline (PBS′), pH 7.40 containing 12 mM Na_2_HPO_4_, 3 mM KH_2_PO_4_, 1.5 mM KCl and 100.5 mM NaCl. Concentration of the K^+^, Na^+^ and Cl^‒^ ions corresponds approximately to that of the human blood serum. Residual citrate content of HSA was removed by repeated ultrafiltration of the protein stock solution, and its concentration was calculated from its UV absorption: *λ*_280 nm_(HSA) = 36,850 M^−1^ cm^−1^ [51]. Stock solutions of WF and DG were prepared as described previously [31]. Samples containing RhCp* and RuCym compounds were incubated for 2 and 24 h, respectively, prior to the measurements, or the reaction kinetic was followed. All samples were prepared in PBS’ and incubated at 25 ± 0.1 °C and measurements were carried out at this temperature.

## ^1^H NMR spectroscopy

^1^H NMR spectroscopic studies were carried out on a Bruker Avance III HD instrument. All spectra were recorded with the WATERGATE water suppression pulse scheme using DSS internal standard. To study the interaction with HSA and the RPMI 1640 components ^1^H NMR spectra were recorded for samples containing 1 mM organometallic compound and 0.5 mM HSA or 1.25-fold diluted RPMI 1640 in PBS’ at 10% (v/v) D_2_O content.

### Spectrofluorometry

Fluorescence spectra were recorded on a Hitachi-F4500 fluorometer in 1 cm quartz cell. Samples contained 1 μM HSA and various HSA-to-metal compound ratios (from 1:0 to 1:20) were used. In the site marker displacement experiments, the HSA-to-site marker (WF or DG) ratio was 1:1 and the concentration of the compounds varied. The excitation wavelengths were 295, 310 or 335 nm depending on the type of the experiment and the emission was read in the range of 310–650 nm. The conditional binding constants (log *K*′) were computed with the computer program PSEQUAD [52] as described in our previous works [31, 53]. The effect of low-molecular-mass (LMM) components on the albumin binding of Ru and Rh compounds was monitored in samples containing 1 μM HSA, 10 μM metal complex and LMM component mixture or only one of these components corresponding to their blood serum concentration: Ser (~ 100 μM), His (~ 77 μM), Met (~ 77 μM) and Cys (~ 33 μM), citrate (~ 99 μM) and lactate (~ 1.5 mM). Samples containing Cys were kept in a strictly oxygen-free atmosphere. Calculations always were based on data obtained from at least two independent measurements. Corrections for self-absorbance and inner filter effect were necessary, since the emitted light was partly absorbed by the compounds. Corrections were carried out according to the following equation [54]:

$$I_{\text{corr}} \, = \,I_{\text{meas}} \, \times \,10^{{\left( {A_{\text{EX}} + A_{\text{EM}} } \right)/2}} ,$$

where *I*_corr_ and *I*_meas_ are the corrected and measured fluorescence intensities, *A*_EX_ and *A*_EM_ are the absorbance values at the excitation and emission wavelengths in the samples, respectively.

### UV–visible spectrophotometry

A Hewlett Packard 8452A and an Agilent Carry 8454 diode-array spectrophotometer were utilized to record the UV–visible (UV–vis) spectra in the interval 190–820 nm. The path length (*l*) was 1 cm. For the reaction kinetic studies, a tandem cuvette with two chambers was used. Measurements were performed at various metal complex concentrations (between 20 and 200 μM), and various protein-to-complex ratios (from 0.02:1 to 2:1) were applied. UV–vis spectra presented in this paper are subtracted by the spectrum of reference albumin sample in all cases in favor of the better interpretation of the results. Reference spectra for the complexes and their components (ligand and organometallic cation) were recorded as well. Excel Solver (Microsoft Office 2016) was utilized for deconvolution of the absorbance spectra.

### Ultrafiltration

Samples were separated by ultrafiltration through 10 kDa membrane filters (Millipore, Amicon Ultra-0.5), in low- and high-molecular-mass (LMM and HMM) fractions with the help of an Eppendorf MiniSpin plus centrifuge (relative centrifugal force ∼ 1500*g*, 5–10 min). Three different types of experiments were carried out: samples contained (1) 50 μM HSA and 50–430 μM organometallic compound; (2) ~ 500 μM organometallic cation and 8–50 μM HSA; (3) reversibility of the protein binding was followed by consecutive washing and filtration steps and then by filtration of samples after addition of 0.5% (m/m) SDS. The concentration of the non-bound compounds in the LMM fractions was determined by UV–Vis spectrophotometry by comparing the recorded spectra to those of reference samples without the protein. When the complex partly decomposed due to the protein binding, the free ligand was also detected in the LMM fraction. In this case, the recorded spectrum was deconvoluted with the use of the molar absorbance spectra of the ligand and metal complex by Excel Solver (Microsoft Office 2016). The HMM fraction was studied in a similar way for some samples as well.

### Capillary zone electrophoresis

Measurements were performed on a G7100 capillary electrophoresis system (Agilent Technologies, Waldbronn, Germany) equipped with a diode-array detector (210–600 nm). For all experiments, fuse silica capillaries of 48 cm total length (50 μm inner diameter) were used (BGB Analytik). Phosphate buffer (20 mM, pH 7.40) was the background electrolyte (BGE). The conditioning process of new capillaries started with HCl (0.1 M) and H_2_O followed by NaOH (0.1 M) and H_2_O for 20 min each and was completed with flushing of the BGE for 40 min. A similar procedure was applied for daily preparation of the capillary although without purging with HCl. To ensure the steady baseline, the capillary was flushed with BGE (2 min) before each run and was rinsed with NaOH (0.1 M; 1.5 min), H_2_O (1.5 min), and BGE (2 min) after each separation. The sample tray and the capillary were kept at a constant temperature of 25 °C. Samples were injected hydrodynamically at 50 mbar for 15 s, and voltage of 30 kV was applied for the separation process producing a current of ca. 44 μA. Sample run time was set to 6 min. Electropherograms were recorded and evaluated by the program ChemStation (Agilent Technologies). The detection wavelength was set to 280 or 260 nm depending on the samples studied. Samples contained ca. 200 μM organometallic compound and various concentrations of HSA (10–100 μM) in PBS′. The peak integrals of the non-bound complex and ligand were used to obtain their concentrations using external calibration. UV–Vis spectra belonging to all peaks were collected as well.

## Results

### Interaction of Rh(η^5^-C_5_Me_5_) compounds with RPMI 1640 cell culture medium

Prior to the investigation of the interactions of biomolecules with organorhodium and organoruthenium compounds, the knowledge of their actual forms at physiological conditions is of critical importance to get adequate conclusions. Dissolving the organometallic precursor ([Rh(η^5^-C_5_Me_5_)(µ-Cl)Cl]_2_ in water at pH 7.40 the triaqua cation [Rh(η^5^-C_5_Me_5_)(H_2_O)_3_]^2+^ and its hydrolysis products [(Rh(η^5^-C_5_Me_5_))_2_(μ-OH)_2_]^+^ and [(Rh(η^5^-C_5_Me_5_))_2_(μ-OH)_3_]^2+^ are formed; their actual quantity depends on the analytical concentration of the dissolved precursor. In the presence of 0.1 M chloride ion, corresponding to the chloride concentration in blood serum, aqua and hydroxido ligands are partly displaced by chloride ions. Namely, a mixture of species is present in the solution. The ruthenium precursor behaves similarly. For this reason, these species (if there is no need for closer definition) are referred generally as RhCp* for Rh(η^5^-C_5_Me_5_) and RuCym for Rh(η^6^-*p*-cymene) species. RhCp* and RuCym hydrolyze in about 23% and 100%, respectively, at physiological conditions at 50 μM concentration [50, 55].

The complexes of the bidentate ligands presented in Chart 1 were obtained by mixing the precursors and the ligands in 0.5:1 ratio in water and by this way the aqua complexes formed (Chart 1). Although, in chloride-containing media, chlorido complexes are present to some extent as well, therefore the leaving group is generally not specified in our complex structures and are referred to as, e.g., RhCp*(dhp) or RuCym(pic). Dhp as an (O,O) donor ligand forms somewhat lower stability complexes with the two organometallic cations compared to the (O,N) donor pic. Dhp complexes of both RhCp* and RuCym dissociate in about 4–5% at pH 7.40 and 50 μM complex concentration at *I* = 0.2 M KCl, while pic complexes are more stable and less than 0.5% dissociate under the same conditions [56–58]. These values may somewhat differ at physiological (100 mM) chloride ion concentration, as chloride ions can compete with the coordinating ligands and suppress the complex formation. RuCym(maltolato) as an example for a low stability complex (29% decomposed under the condition vide supra) was also selected [59], and complexes of picolinate derivatives 6-Mepic and 2-quinaldic acid formed with RhCp* characterized by higher lipophilicity than the pic complex were involved in our investigations as well [60] (constants regarding the complex stability, chloride affinity and lipophilicity data are listed in Table S1).

In vitro cytotoxicity studies are always carried out in cultured media, which are produced in different compositions according to the actual use. Some compounds are essential and universal components of these mixtures. All kinds of minimum essential media (MEM or Eagle’s MEM) contain phosphate and bicarbonate buffered saline, inorganic ions Ca^2+^, Mg^2+^, K^+^, Na^+^, essential l-amino acids (histidine, (iso)leucine, lysine, methionine, phenylalanine, threonine, tryptophan, valine) and some non-essential l-amino acids (e.g., arginine, cystine, tyrosine). While phosphate was proven to be a ‘safe’ buffer for these organometallic compounds in our preliminary measurements, hydrogencarbonate and amino acids are potential ligands of organorhodium and organoruthenium cations and can be effective competitors of the original ligand set in this kind of complexes [61–64]. The medium applied here was RPMI 1640, containing 20 alpha amino acids, vitamins, glucose, glutathione and phenol red.

**Fig. 1:**
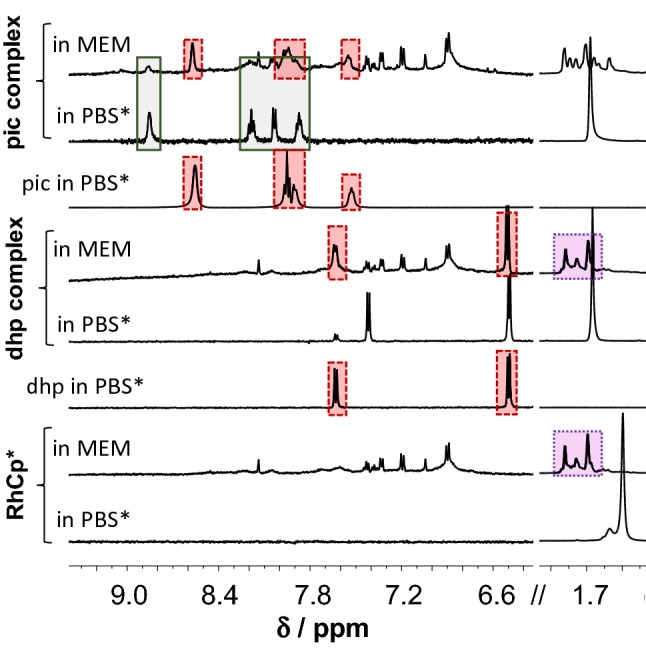
Stability of the organometallic ion RhCp* and its pic and dhp complexes in RPMI 1640 medium. Peaks in solid, dotted and dashed frames denote the original complex, the forming complexes without the original ligand, and the unbound ligands, respectively [*c*_complex_ = *c*_lig_ = 1 mM, in RPMI 1640 or PBS’ medium, 10% D_2_O, 25 °C]

^1^H NMR spectra in Fig. 1 show the interaction of organorhodium ion and its dhp or pic complexes with RPMI 1640 components. The original RhCp*(dhp) complex is completely dissociated, dhp and (most probably) coordinating water as well are replaced by RPMI 1640 components, which results in the appearance of the same peak set for C_5_Me_5_ methyl protons like in case of RhCp* itself, and no mixed-ligand complex formation can be observed. A similar result was obtained with related complex RhCp*(acetylacetonato) in our former work [65]. The pic complex reacts somewhat differently: about 75% of the ligand is liberated here as well, but the rest is coordinated to the metal ion. Splitting and shifting of the C_5_Me_5_^−^ proton signal, however, suggest mixed-ligand complex formation with medium component(s). In cytotoxicity measurements, the tested compounds are dissolved typically in micromolar concentrations in cell medium, and more pronounced decomposition of the original complexes is expected under these conditions. Consequently, cytotoxicity measured for lower stability complexes (e.g., the dhp or acac complexes) did not exceed the sum of efficacy measured for organorhodium ion (coordinated to medium components) and the ligand (if it is active) separately [56, 65].

Cultured media often contain ca. 10% heat-inactivated fetal bovine serum. Serum proteins including albumin as potential reaction partners for metal complexes are present in these samples as well. Studying the interaction of these organometallic compounds with proteins, especially with human serum albumin is important in other aspects as well, which are discussed in the next sections in detail.

### Binding of (Rh(η^5^-C_5_Me_5_) and (Ru(η^6^-*p*-cymene) to human serum albumin

Binding of anticancer metallodrugs to HSA is of considerable interest, as it may have a significant effect on the biodistribution, toxicity and side effects. Moreover, HSA- and HSA-bound drugs can accumulate in malignant and inflamed tissues such as solid tumors as a consequence of the enhanced permeability and retention effect, which can be exploited for tumor targeting as well [20]. Organometallic fragments RhCp* and RuCym (Chart 1) per se exert no antitumor activity, however a deeper knowledge on their interaction with albumin is a mandatory prerequisite for understanding the binding mechanism of their complexes to this protein.

The binding mode and binding strength of the organorhodium ion (without coordinated ligands) on HSA were investigated in our former work [49]. However, albumin binding of organoruthenium ion was not characterized adequately in solution, since only its binding at the hydrophobic site I (IIA subdomain) was reported [57]. This section provides a detailed kinetic, quantitative and qualitative description on the HSA binding of RhCp* and RuCym species.

**Fig. 2:**
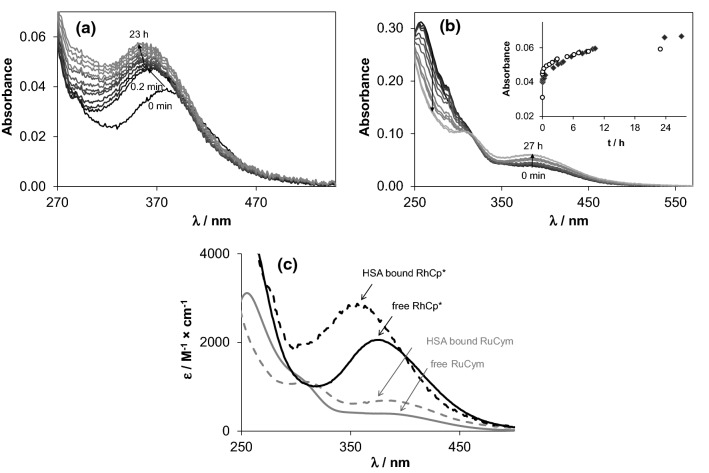
Albumin binding kinetics of the organometallic ions RhCp* (**a**) and RuCym (**b**) followed by UV–Vis spectrometry, and calculated molar absorbance (*ε*) spectra of the HSA-free and HSA-bound forms of RhCp* and RuCym (**c**). Inset shows the absorption changes at 350 nm for RhCp* (empty circle) and at 390 nm for RuCym (filled diamond), respectively. Spectra depicted here are subtracted by the spectrum of HSA [*c*_Rh_ = 20 µM and *c*_HSA_ = 10 µM (**a**); *c*_Ru_ = 100 µM and *c*_HSA_ = 33 µM (**b**); PBS’; 25 °C]

Both organometallic cations displayed considerable changes in their UV–vis spectra in Fig. 2 in the presence of HSA. RhCp* showed a bi-phasic binding profile, where a fast (~ 5 min) process is followed by a much slower one (~ 24 h). Based on our ultrafiltration, and CZE studies, the binding is completed in the first few minutes, and the subsequent spectral changes in Fig. 2a can be attributed most probably to structural rearrangement of the coordination sphere around the metal ion. On the contrary, binding of RuCym to the protein takes place about 24 h (Fig. 2b) confirmed by ^1^H NMR spectroscopic, ultrafiltration and CZE time-dependent measurements. The observed remarkably different binding rates of the two organometallic cations partly can be explained by their different solution speciation at pH 7.40. RuCym is present in 100% as dimeric hydroxido species [(RuCym)_2_(μ-OH)_3_]^+^, which is known to be kinetically more inert compared to the aqua cation, while in case of RhCp* there is ca. 55% non-hydrolyzed [Rh(η^5^-C_5_Me_5_)(H_2_O)_3_]^2+^ in solution under this condition [50, 55, 66]. Furthermore, as a result of the markedly increased *trans* effect of the anionic pentamethylcyclopentadienyl ligand in comparison to the neutral *p*-cymene ligand, typical substitution rates for RhCp* complexes are several orders of magnitude higher (~ 10^2^–10^3^ s^−1^ for H_2_O exchange in monoaqua complexes) than for analogous Ru(II)-arene anticancer compounds (~ 10^−3^–10^−1^ s^−1^) [67, 68]. In subsequent experiments, samples containing RuCym or RhCp* were always measured after 24 h and 1 h waiting time, respectively.

It should be mentioned that commercially available lyophilized HSA may contain residues of citric acid as anticoagulant additive of the original blood stock. The presence of citrate can be detected by ^1^H NMR spectroscopy (Fig. S1). Citrate is a coordinating ligand of RuCym [69] (and probably of RhCp* as well), consequently peaks of the citrate complexes could be observed in ^1^H NMR spectra of citrate-contaminated protein samples (Fig. S1). Furthermore, we have found that presence of citric acid increases the binding rate of RuCym towards albumin about four to fivefold. Therefore, citrate content of HSA stocks was always checked by ^1^H NMR spectroscopy and, if it was necessary, HSA stock solutions were purified by ultrafiltration.

**Fig. 3:**
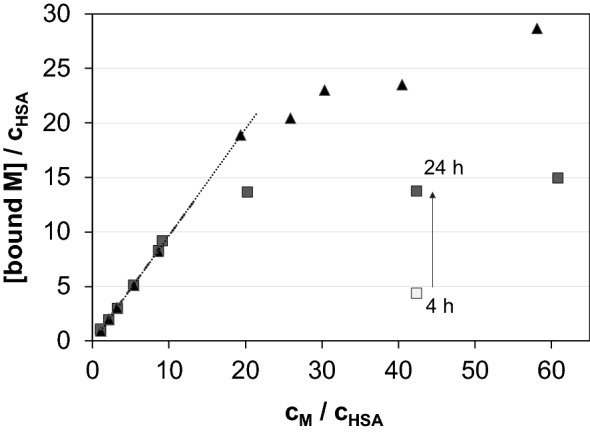
Binding capacity of HSA for RhCp* (filled triangle) and RuCym (filled square) determined by ultrafiltration–UV–Vis measurements. Empty symbol (empty square) denotes the bound equivalents of RuCym after 4 h incubation time. Slopes of the fitted lines are: *s* = 0.971 (*R*^2^ = 0.999) for RhCp* and *s* = 0.969 (*R*^2^ = 0.997) for RuCym [*c*_M_ is varied at < 10 *c*_M_/*c*_HSA_ (50–430 μM) and *c*_M_ ≈ 600 uM at > 10 *c*_M_/*c*_HSA_; incubation time: 1 h for Rh and 24 h for Ru compound; in PBS’; 25 °C]

HSA is able to accommodate 8–9 eq RhCp* as it was reported in our former work [49], although these numbers do not seem to be the upper limit of available binding sites for RhCp*. The results of ultrafiltration studies presented in Fig. 3 reveal nearly quantitative binding of even 20 eq of cations on HSA and a maximal number of ca. 24 ± 3 RhCp* can be bound by the protein. Practically quantitative binding of RuCym can be observed up to 9 eq of metal ion, and binding capacity of HSA is saturated with ca. 14 ± 1 eq of RuCym.

Both RhCp* and RuCym may bind to HSA in a coordinative manner, but a striking difference is seen in the number of coordination sites. Coordination of His imidazole nitrogen is suggested by Sadler et al. in the interaction of HSA with RM175 complex [38]. HSA contains 16 His residues as possible coordination sites, however, it is less, than the maximal number of bound RhCp*, and some of these histidine nitrogens may be not easily accessible for the organometallic cations. Interaction with sulfur donor side chains of Met and Cys can be assumed as well based on the works of Briš et al. and Hu et al. [70, 71]. Free thiol groups of small ligands Cys, glutathione and *N*-acetyl-cysteine show high affinity towards RuCym(PTA) complexes [70], while the single free thiol group in HSA (Cys-34) was reported to undergo oxidation during its interaction with [RuCym(ethylenediamine)Cl]^+^ resulting in the formation of sulfinato complex without dissociation of the ethylenediamine ligand [71]. In the same work surface-exposed His-128, His-247, His-510 and Met-298 are also denoted as coordination sites of Ru(arene) complexes [71]. X-ray single crystal structures reported for protein–RuCym complex adducts often show coordination of amino acids His or Glu to the metal center [40–46], and coordination of Lys, Arg, Asp and Cys side chains occurs as well [41, 46, 47]. Coordination of one or two of these side chains is typical, and the loss of *p*-cymene ligand can be detected in some cases as well [40, 41, 47]. Apo-ferritin coordinated [(N_His_,N_His_,O_Glu_)] is the only reported structure, where coordination sphere of RuCym is saturated by the donor groups of the protein [41]. To the best of our knowledge, there is only one structure reported on protein-coordinated RhCp*; here two organorhodium fragments are bound to His-112 and His-121 imidazoles, respectively, in streptavidin [48]. Namely, mono or bidentate coordination seems to be feasible in HSA, while binding of HSA donor groups in tridentate mode is most probably sterically less favored. Involvement of S-donor Cys-34, Met (6 residues in HSA); O-donor glutamate (62), aspartate (36) or N-donor lysine (59) or arginine (24) side chains is feasible in binding of high excess of RuCym and RhCp*.

**Fig. 4:**
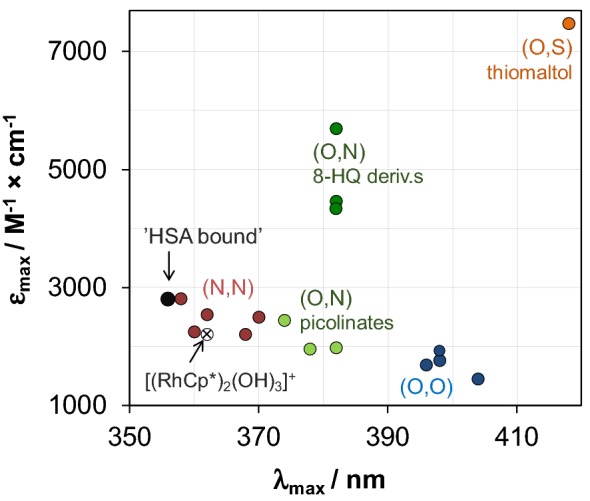
Correlation diagram for the *λ*_max_ and *ε*_max_ values of various bidentate [RhCp*(L)Cl] complexes and dimeric hydroxido species [(RhCp*)_2_(μ-OH)_3_]^+^ and HSA-bound RhCp* at 1:1 HSA-to-Rh ratio (correlation diagram for the aqua complexes ([RhCp*(L)(H_2_O)]) are presented in Fig. S3; see exact values for each complexes in Table S2)

It is difficult to get a clue on the binding environment of the organometallic cations in solution; however, UV–vis spectra can provide some information about the coordination sphere(s) forming around the organometallic cations inside the protein. Individual molar spectrum of the ‘albumin-bound’ forms can be obtained both in steady-state UV–vis measurements (Fig. 2c) and via examining albumin-bound fraction of ultrafiltered or electrophoretically separated samples (Fig. S2). As Fig. 2c shows, dinuclear [(RuCym)_2_(μ–OH)_3_]^+^ transforms into a typical mononuclear complex bound form, and two charge-transfer (CT) bands develop with *λ*_max_ = 310 nm (*ε*_max_ = 1100 M^−1^cm^−1^) and 382 nm (*ε*_max_ = 670 M^−1^cm^−1^), which did not depend significantly on the Ru-to-HSA ratio applied. Notably, the recorded spectra did not indicate the loss of *p*-cymene ligand from the Ru(II) center (see details in Refs. [72, 73]). More detailed qualitative information can be obtained on the albumin-bound form of RhCp* (Fig. 2c), where *λ*_max_ = 356 nm and *ε*_max_ = 2800 M^−1^ cm^−1^ values of the forming protein complex can be compared to those of various complexes of bidentate ligands studied formerly in our research group (Table S2) [49, 50, 56, 60, 65, 72, 74, 75]. The correlation diagram presented in Fig. 4 (and Fig. S3) illustrates well the effect of each donor groups on the *λ*_max_ and *ε*_max_ values: *λ*_max_ increases while *ε*_max_ decreases tendentiously by changing the donor sets from (N,N) to (O,N) and then to (O,O). Additionally, coordination of the chlorido ligand in the third position induces red shifted *λ*_max_. According to the correlation diagram, spectral parameters of HSA-bound RhCp* are close to the (N,N)(Cl)-type complexes (like complexes of ethylenediamine, bpy and their derivatives) raising the possibility of (N_imidazole_,N_amide_)-type coordination. These spectral parameters are practically unaltered between 1:1 and 8:1 Rh-to-HSA ratio, but increasing the Rh excess from 8:1 to 20:1 results in a gradual shift to *λ*_max_ = 373 nm and *ε*_max_ = 2270 M^−1^ cm^−1^, indicating the involvement of other types, probably (O,N), (O,O) and/or (O) donor sites into the binding of RhCp* on HSA as well. Another explanation can be the appearance of monodentate N-donor binding sites, which consequently resulted in red shifted *λ*_max_ [76] as well.

Reaction rates of coordinating ligands with RuCym are strongly affected by the type of the donor atoms. Complex formation with (O,O) or (O,O,O) donor ligands is a fast process at physiological pH, while (N) and (N,N,N) donor ligands chelate the RuCym rather slowly (hours to days) [59, 69, 76, 77]. Reaction rate with (O,N) donor bearing ligands mainly depends on the actual protonation state of the given donor atom, thus it also depends on the actual pH, and the interaction may take place from few minutes to several hours [57, 72]. In this view, slow coordination of protein (N)-donor group(s) to RuCym can be assumed.

Spectrofluorometric studies were carried out to investigate the binding of RuCym at hydrophobic binding pockets site I (in subdomain IIA) and site II (in subdomain IIIA). Binding of RhCp* was formerly investigated by Trp-214 quenching (at site I) and site marker displacement experiments using WF and DG to follow interaction at sites I and II, respectively. High-affinity binding and fast binding rate (few min) were found for RhCp* at both sites [49]. RuCym displays slow binding at site I, as it was observed in the global binding studies (vide supra) as well and exerted affinity at binding pocket I comparable to that of RhCp* (see Table 1). RuCym additionally interacts with free DG and quenches its fluorescence. Therefore, the binding at site II is observed but data can not be quantitatively evaluated. Since aqua complexes are partly chlorinated and can hydrolyze at pH 7.40, all protein binding constants determined here are regarded as conditional stability constants and valid only under the given conditions. Considering the extremely high number of binding sites on HSA (which cannot be included in the calculations), the binding constants calculated here reflect the competitiveness of sites I and II against the other sites together with their affinity for the free metal compounds. Namely, higher constants could be expected if the binding event at isolated sites could be studied. Slow binding of RuCym at site I indicates the involvement of (N) donor His-242 lining binding site I. Most probably RhCp* binds to the same His residue here, while the nature of coordinating donor group at site II is in question, since no His can be found among the residues evolving in this pocket [78].

**Table 1:** Conditional binding constants (log *K*′) of the compounds at binding sites I and II of HSA determined by spectrofluorometric quenching (log *K*_Q_′) and marker displacement log *K*_WF_′ and log *K*_DG_′ measurements [pH 7.40 (PBS′); 25 ºC]

	Site I		Site II
	Trp-214 quenching	WF competition	DG competition
RuCym^a^	5.60^b^	–	–
	5.7 ± 0.1	5.9 ± 0.1	nm^c^
Complexed by			
Maltol	5.4 ± 0.1	5.8 ± 0.1	5.7 ± 0.1
dhp	5.7 ± 0.1	5.7 ± 0.1	5.5 ± 0.1
pic^d,e^	4.3 ± 0.1	4.2 ± 0.1	4.2 ± 0.1
RhCp*^f^	5.8	6.1	5.8
Complexed by			
dhp^f^	5.9	6.2	5.8
pic^e^	5.1 ± 0.1	5.6 ± 0.1	5.1 ± 0.1
6-Mepic	5.4 ± 0.1	5.9 ± 0.1	5.6 ± 0.1
2-Quinaldic acid	5.5 ± 0.1	5.8 ± 0.1	5.5 ± 0.1
Ethylenediamine^f^	Weak	Weak	Weak
bpy^f^	Weak	Weak	Weak

^a^log *K*_Q_′ = 5.25 reported for the related organometallic cation [Ru(η^6^-toluene)(H_2_O)_3_]^2+^ [57]

^b^Reported in Ref. [65]

^c^Could not be calculated because RuCym interacts with DG

^d^log *K*_Q_′ = 4.16 reported for the complex [Ru(η^6^-toluene)(pic)(H_2_O)]^+^ [57]

^e^Reported binding affinities of pic: log *K*_Q_′ = 4.18, log *K*_WF_′ = 4.05, log *K*_DG_′ = 3.65 [79]

^f^Reported in Ref. [49]

### Interaction of Rh(η^5^-C_5_Me_5_) and Ru(η^6^-*p*-cymene) complexes of dhp, pic and some related ligands with human serum albumin

Selection of the complexes was mainly governed by their well-characterized and somewhat different solution chemical properties and not by the otherwise poor cytotoxic efficacy of the complexes. These selected complexes, however, are good models of in vitro active compounds, e.g., (O,O) donor flavone and naphtoquinone-type complexes of Ru(η^6^-*p*-cym) or (O,N) donor 8-hydroxyquinoline or Schiff-base complexes of both metal ions [72, 80–85]. It was shown previously that interaction of the studied complexes with HSA is possible, both by loss of the bidentate ligand or by the loss of only chlorido leaving group [49]. The former one is considered as dissociative binding as the original ligand becomes unbound, while the latter one is referred here to as associative binding, i.e., binding of the ‘non-dissociated’ complex to the protein. Complex stability seems to play a key role in the type of the protein binding [49]. This way, it is useful to take a look at the reported solution chemical behavior of the studied complexes, since they could have a profound effect on their protein binding (Table S1). As it was mentioned (vide supra), the complex stability follows the following trend: maltol < dhp < picolinates at pH 7.4 (Table S2).

First of all, global binding kinetics of the complexes towards albumin was studied by UV–Vis spectometry. RhCp* complexes interacted relatively fast with HSA and reaction rates did not depend on the complex-to-HSA ratios (Fig. S4a and b). The dhp and pic complexes were bound within 10 and 60 min, respectively, to the protein. RuCym(dhp) at the same time displays a completely different behavior, a bi-phasic binding profile is characteristic with a fast, 10 min process and a much slower > 24 h phase. The equilibrium state could not be achieved even after 2 days, but longer studies are not reasonable from the physiological point of view; therefore subsequent samples were measured after 24 h waiting time. Samples with RuCym(pic) ultrafiltered after 4 h and 24 h indicated binding of the complex in a fairly similar extent in both cases without the release of pic. Based on these findings, the Rh and Ru samples were incubated for 2 h and 24 h, respectively.

**Fig. 5:**
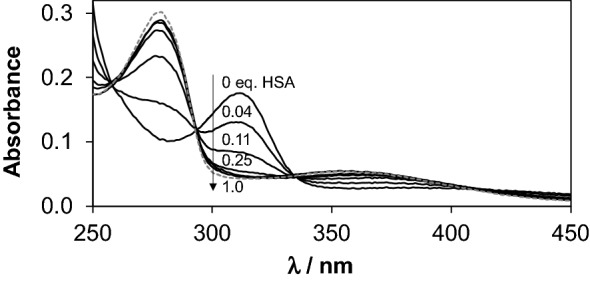
UV–Vis absorption spectra of the HSA‒RhCp*(dhp) system recorded at different HSA-to-complex ratios and summed spectrum (gray dashed line) calculated for system containing free dhp and HSA-bound RhCp*. Spectra are subtracted by the corresponding absorbance spectrum of HSA [*c*_complex_ = 20 μM, *c*_HSA_ = 0–20 μM; in PBS’, 25 °C. See further details in the “Experimental” Section]

**Fig. 6:**
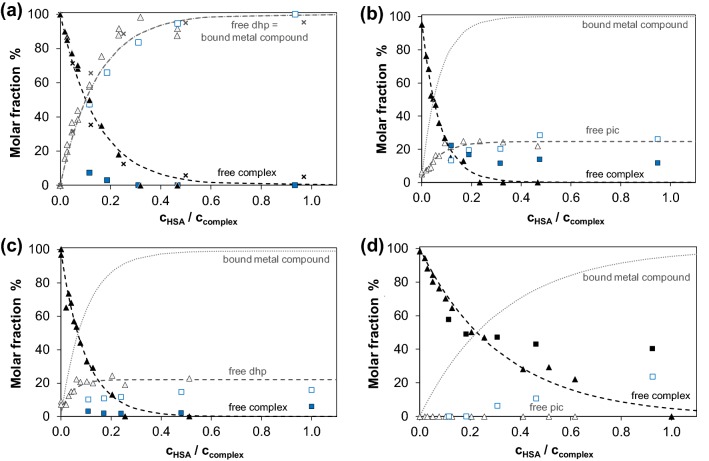
Molar fractions obtained for albumin-bound and unbound compounds in the HSA–RhCp*(dhp) (**a**), –RhCp*(pic) (**b**), –RuCym(dhp) (**c**) and –RuCym(pic) (**d**) systems calculated on the basis of UV–Vis titration (×), CZE (filled triangle, empty triangle) and ultrafiltration–UV–Vis (filled square, empty square) measurements. Dashed curves were fitted for CZE data, and fractions of bound metal compound (dotted line) were calculated on this basis (*x*(bound metal comp.) = 100 – *x*(free complex). Molar fraction (%) is given as: [compound]/*c*(metal complex) × 100 [*c*_complex_ = 20 μM (UV–vis), 200 μM (CZE), 0–430 μM (ultrafiltration); PBS′, 25 °C; see more details in the “Experimental” Section]

Mainly dissociative binding of RhCp*(dhp) was reported based on ultrafiltration and ^1^H NMR experiments in our former work. Our new results obtained by UV–vis and CZE measurements were compared to that of previous ultrafiltration data. Figure 5 demonstrates the effect of increasing amounts of HSA on the UV–Vis spectra of RhCp*(dhp). Decomposition of the complex and appearance of free dhp and albumin-bound RhCp* were observed. According to former investigations, dhp itself does not interact with HSA [79]. Isobestic points in Fig. 5 indicate the predominance of only one kind of equilibrium process. The original recorded spectra could be deconvoluted to the spectra of HSA, HSA-bound RhCp*, free dhp and free RhCp*(dhp) according to the hypothesized equilibrium RhCp*(dhp) + HSA ⇌ RhCp* − HSA + dhp. Deconvolution of the spectra gave a fairly good fit even at high complex excess (see Fig. S5). The CZE method turned out to be a particularly useful tool for both quantitative and qualitative evaluations of the interaction. Samples for CZE measurements were prepared in PBS′, but 20 mM phosphate (pH 7.40) was chosen for BGE to avoid peak splitting and broadening caused by the fast equilibration of aqua and chlorido complex forms in the electrophoretic capillary. Accordingly, the aqua complexes [M(ligand)(H_2_O)]^+^ (M=RhCp* or RuCym) were detected in CZE experiments. Each species separated well in the capillary: aqua complexes are + 1 charged, dhp is neutral, pic is present in its anionic form and HSA is negatively charged under the given conditions. Calculated fractions computed from the peak areas of the recorded electropherograms for unbound complex/ligand and then for the protein-bound complex are depicted in Fig. 6a. These fractions are in fairly good agreement with the results of UV–vis studies. Interestingly, ultrafiltration data revealed much lower quantities of free RhCp*(dhp) compared to CZE and UV–vis experimental data indicating the binding of non-dissociated RuCym(dhp) on HSA as well. The ultrafiltration experimental setup, however, differs from the other two methods basically, since the metal complex concentration was varied here in contrast to CZE and UV–Vis studies, where the complex concentration was kept constant, and that of albumin was varied. Careful analysis of the absorbance spectra of the ‘protein peak’ in the electropherograms confirms the binding of RhCp* without the dhp ligand on HSA (Fig. S6). Therefore, stacking of the complex to the filter is probable in ultrafiltration measurements resulting in overestimated quantities of bound RhCp*(dhp). Free fractions of dhp (which is equal to the bound fraction of organometallic ion) are well comparable in all three methods.

**Fig. 7:**
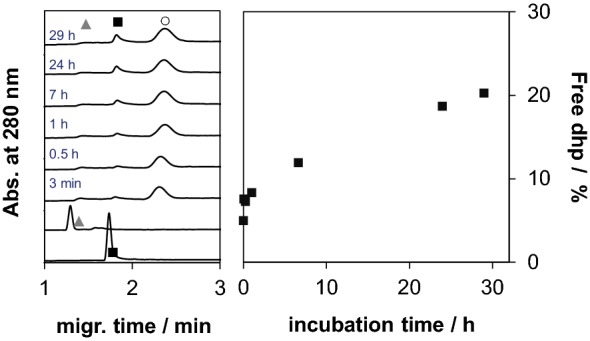
Electropherograms recorded for HSA–RuCym(dhp) 1:10 system after various incubation times and electropherograms of the complex and the ligand alone; symbols denote the free complex (gray triangle), free dhp (black square) and HSA (white circle) peaks. Right side: calculated fractions of dhp released as a function of time based on CZE (black square) measurements [*c*_complex_ = 215 μM; PBS’ buffer, 25 °C]

The interaction of RuCym(dhp) with HSA appears to be basically different from that of RhCp*(dhp). The bi-phasic binding profile mentioned before is due to the fast binding of the complex on HSA, and then slow release of dhp took place in the second phase as it is shown in Fig. 7. This process did not lead to the complete release of dhp, ca. 20% of the ligand was liberated after 24 h incubation in CZE experiment in a sample with 1:10 HSA-to-complex ratio. Namely, release of dhp occurs here as well, however, this phenomenon is more likely a kinetically governed process and only 20–25% free dhp could be detected even after 24 h by both spectroscopic and separation techniques (^1^H NMR, CZE, ultrafiltration) at various HSA-to-complex ratios. Kinetic curves for this binding event were recorded at various HSA-to-complex ratios by UV–vis, which show increasing rates of dhp release at higher HSA concentrations (Fig. S4c).

**Fig. 8:**
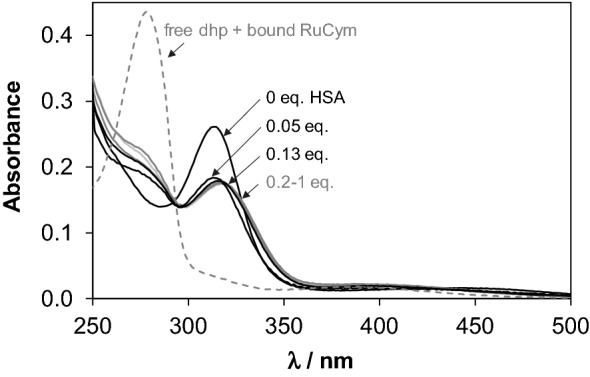
UV–Vis absorbance spectra of RuCym(dhp) in the absence and presence of various eq of HSA indicated in the figure. Dashed spectrum was calculated as sum of free dhp and HSA-bound organoruthenium ion. Spectra are subtracted by the spectrum of HSA [*c*_complex_ = 29 μM, PBS′, 25 °C]

Notably, at lower HSA concentrations, the free portions of the RuCym(dhp) complex obtained in ultrafiltration experiments are almost negligible compared to those of in CZE samples (Fig. 6c). In other words, ultrafiltration predicts remarkably high, ca. 98% binding of the metal compounds (intact complex and organometallic ion together) at 1:0.12 HSA-to-complex ratio, while CZE provides only ca. 76%. UV–vis spectra in Fig. 8 recorded for the RuCym(dhp)–HSA system show only moderate absorbance increase in the wavelength range where free dhp absorbs light (*λ*_max_ = 278 nm). This refers to the predominant binding of non-dissociated RuCym(dhp), and shows high similarities to those obtained for the albumin bound compound fraction in the CZE studies (Fig. S6c). Spectra are not altered between 300 and 550 nm at 0.2–1 eq of HSA, namely fivefold excess of metal complex seems to be bound quantitatively to albumin, and even at 20-fold excess (0.05 eq. HSA) spectra show stronger similarities to the protein-bound form than to the spectrum of the complex not bound to the protein (Fig. 8). More detailed evaluation of the spectra is not possible, since both the Ru complex and the RuCym bind parallel on HSA, the individual spectrum of the HSA-bound complex is unknown. In all, CZE and UV–vis data suggest the presence of some free RuCym(dhp) in samples containing less than 0.2 eq of HSA, while negligible extent of free complex was registered in ultrafiltration experiments. Complex might stick on the filter in the latter case, although both RhCp* and RuCym complexes of dhp filtered alone displayed only 9% and 6% loss of compound in the filtered samples, respectively. RuCym(maltolato) complex displayed a similar behavior like RuCym(dhp) in ultrafiltration experiments, except this complex bound considerably slower, as a results of the decomposition of the complex in ca. 29% forming hydrolyzed [(RuCym))_2_(μ-OH)_3_]^+^ species under the experimental conditions.

**Fig. 9:**
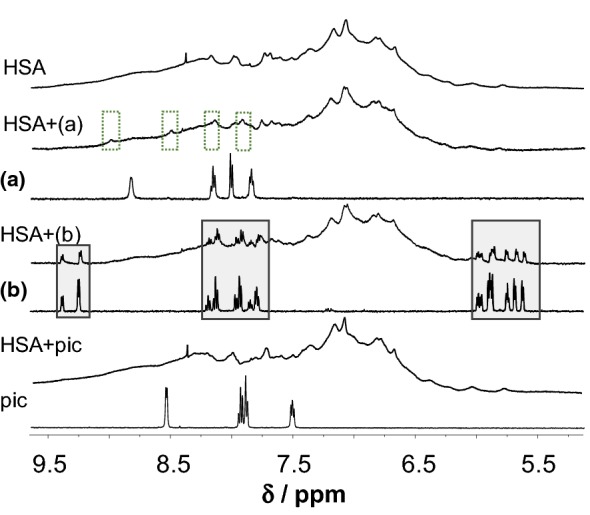
^1^H NMR spectra recorded for HSA and HSA–RhCp*(pic) (**a**) or RuCym(pic) (**b**) or pic systems (dotted frame denotes HSA-bound pic complex, solid frame shows non-bound complex signals) [*c*_complex_ = 1 mM, *c*_HSA_ = 0.5 mM, PBS′, 10% D_2_O]

Taking a look at Fig. 6/b, the interaction between HSA and RhCp*(pic) seems to take place according to the same scenario that was seen in case of RuCym(dhp). At the same time, there are important differences between the two systems: (i) binding of RhCp*(pic) proceeds much faster and (ii) pic itself can bind to the protein via intermolecular bonding [79]. The binding of pic to HSA is relatively weak; two low-affinity binding sites were identified formerly by spectrofluorometric measurements (see Table 1). Ultrafiltration studies confirmed the binding of at least one pic on HSA as well [79]. This way, the detected free pic in Fig. 6b does not correlate directly with the dissociated amount of RhCp*(pic) upon binding to HSA. Ultrafiltration and CZE studies provide similar fractions of free pic, while fractions of free complex differ somewhat at higher HSA-to-complex ratios. ^1^H NMR spectra in Fig. 9 indicate predominant binding of the metal species in complexed form at 1:0.5 complex-to-HSA ratio and C_5_Me_5_^−^ proton signals are different from those of the bound organometallic cation (not shown here). In the case of RuCym(pic), signals of unbound complex were predominant in ^1^H NMR spectra of Fig. 9 in the presence of albumin after 24 h incubation time. CZE and ultrafiltration experiments confirmed lower affinity binding of this complex, and non-dissociated complex is assumed to bind mainly on the protein. UV–Vis studies showed minimal spectral changes in the presence of HSA, implying small, or no rearrangement in the coordination sphere of Ru(II) ion. Combining the results of the four techniques applied in this work, we can conclude that binding of the pic complex is thermodynamically less favored than in case of the other metal complexes studied. Similar tendencies were reported for RhCp*(ethylenediamine) and RhCp*(bpy) complexes in our former work, which were bound to a lower extent and retained their original structure [49].

**Chart 2:**
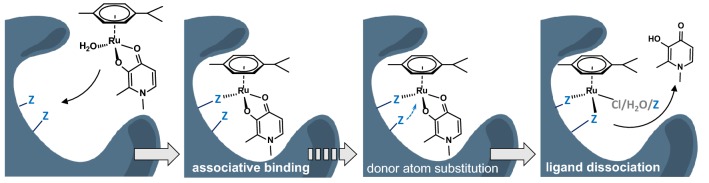
Presumed mechanisms of binding of the studied metal complexes on HSA. The initial step is monodentate coordination of a HSA donor atom displacing the leaving group (H_2_O here) in the complex. Dissociation of the bidentate ligand (dhp) is possible if a second donor atom of HSA is in coordinating position and is strong enough to replace the ligand. The binding of RuCym(dhp) is shown here, Z = donor atom of HSA

Distribution diagrams in Fig. 6 provide further insight into the binding mechanism of the complexes. For example, stabilization of free ligand fraction at ca. 20% for RuCym(dhp) and 25% for RhCp*(pic) in the presence of increasing amounts of HSA indicates an associative ligand exchange mechanism as shown in Chart 2, that was ascertained for RuCym(dhp). Namely, metal complexes bind coordinatively (covalently) to the protein by displacement of the aqua (or chlorido) leaving group by protein donor atoms, and release of ligand at particular sites occurs while the protein coordinates with additional function(s) to the organometallic fragment to achieve thermodynamic equilibrium. The equilibrium state is strongly affected by the solution stability of the original metal complex at pH 7.4 and the stability of the forming HSA–metal ion adduct. This conception can be extended for albumin binding of RuCym(pic) and most probably for RhCp*(dhp) as well. Although the organometallic cation is ultimately bound in case of this complex, the maximal number of binding sites is much lower, about 8 ± 1 compared to the 24 ± 3 available sites in the case of RhCp* (Table 2). This finding confirms the binding as metal complex (bearing clearly less available binding sites on HSA) and subsequent release of the ligand. Generally, the estimated number of available binding sites for the complexes is less compared to those of the organometallic cations (see Table 2). Thermodynamic control in ligand release is seen well via the example of RhCp*(dhp) and RhCp*(pic), where pic complex, owing higher stability (Table S1), shows much less tendency to decompose upon binding to HSA-binding sites, while the dhp complex dissociates completely in the presence of HSA. On the other hand, HSA-induced decomposition of the Ru complexes seems to be hindered kinetically, and observed fractions of free ligand (after 24 h incubation) represent only a temporary picture on ligand dissociation, which may take several days for the dhp complex and even more for the pic complex.

**Table 2:** Bound equivalents of the Rh and Ru compounds per HSA calculated on the basis of CZE measurements at HSA-to-compound ratios of 1: > 20

HSA-to-compound ratio	1: > 20^a^		
Bound form	Complex	Metal ion	Σ
RhCp*	–	24 ± 3^b^	–
dhp complex	0	8 ± 1	8 ± 1
pic complex	nm^c^	nm^c^	12 ± 1
RuCym	–	14 ± 1^b^	–
dhp complex	5 ± 0.7	4 ± 0.4	8 ± 0.6
pic complex	nm^c^	nm^c^	4 ± 0.6

For metal complexes bound forms of the original complex (complex) and of the organometallic cation (metal ion) and their sum (Σ) are listed as well [25 °C, pH  7.40 (PBS′)]

^a^Calculated values are based on at least four measurements

^b^Calculated on basis of ultrafiltration measurements, see details in the Experimental Section

^c^Not measurable. Ligand pic binds on HSA as well, therefore values of bound complex and metal ion cannot be estimated, only the sum of them

Interaction of the metal complexes in the hydrophobic pockets of HSA was investigated as well via the spectrofluorometric technique. Kinetic studies showed fast (~ 10 min) binding of RhCp*(dhp), RhCp*(pic) and RuCym(dhp) at site I (IIA subdomain) corresponding well to the results of global binding studies. Computed binding constants of the metal complexes obtained by Trp-214 quenching and site marker probe displacement studies at sites I and II are listed in Table 1. In the case of lower stability complexes RhCp*(dhp), RuCym(maltolato) and RuCym(dhp), these constants are fairly similar to those of estimated for the corresponding organometallic cation, while elevated complex stabilities lead to lower binding affinities at sites I and II. There is no reasonable difference between the binding affinities of the RhCp* complexes formed with picolinate derivatives, pic, 6-Mepic and 2-quinaldic acid (Chart 1) The more lipophilic character of the 2-quinaldic acid complex has a minimal effect on the binding strength. Binding affinities of RhCp*–picolinate complexes at the hydrophobic binding sites are about one order of magnitude higher compared to those of RuCym(pic) and [Ru(η^6^-toluene)(pic)(H_2_O)]^+^ [57].

Summarizing, a coordinative interaction of the studied complexes with HSA was found. Low stability and kinetically labile complexes (e.g., RhCp*(dhp)) decompose to a high extent upon binding to the protein, while RhCp*-picolinate-type ligand complexes of higher stabilities and the more inert RuCym complexes dissociate to a less extent. Similar tendencies were reported for Ru(arene) complexes interacting with proteins due to the obtained results of X-ray diffraction method [42, 43, 86]: the interaction of presumably low stability complexes formed with (O,O) donor 3-hydroxy-2-pyridone derivative ligands resulted in ligand release and two organoruthenium cations were coordinated by amino acid side chains (His-79 and His-106, Glu-102) of a histone protein [43]. At the same time, high-stability complexes of RuCym formed with ethylenediamine ((N,N) donor) and pyridine-thioamide type ((N,S) donor) ligands did not decompose and complexes are coordinated monodentately by His or Glu amino acid side chains [42, 86].

### Reversibility of HSA binding and the effect of low-molecular-mass serum components

**Fig. 10:**
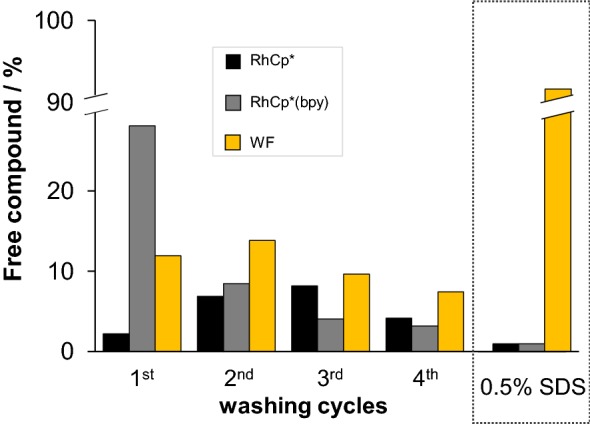
Free fractions of RhCp*, RhCp*(bpy) and WF in 1:1 HSA-to-compound systems followed via ultrafiltration–UV–Vis after consecutive washing cycles or after denaturation of HSA (0.5% SDS) [*c*_comp_ = *c*_HSA_ = 200 μM, PBS′, 25 °C]

The reversible binding of a compound on serum proteins may have a profound effect on its pharmacokinetics and accumulation in solid tumor tissues. Thus, reversibility is a key feature, protein-bound and free fractions of a drug should be in equilibrium, otherwise irreversibly bound fractions can easily decrease the effectiveness of a drug. First, the release of the bound organometallic cations, RhCp*(bpy) and WF, as a high-affinity binding ligand of HSA [87], was tested by consecutive ultrafiltration experiments. Figure 10 shows free fractions of each compound. After the first washing cycle, RhCp*(bpy) was bound to HSA exclusively in an associative manner and to a lower extent compared to RhCp* in accordance with our former results [49]. In the next cycle, liberated portions of both organometallic compounds remain under 10%, namely, binding of the organometallic compounds seems to be at least partly reversible reaction, however, summed fraction of free RhCp* (25%) is ca. half of free WF (47%) after the fourth washing step.

Another interesting question is the fate of bound compounds after denaturation of the protein, e.g., after entering an acidic vesicle in cells. It was tested in a model experiment by the addition of SDS to the reaction mixtures 15 min prior to the filtration. SDS induces unfolding of the protein leading to the loss of specific sites providing intermolecular interactions for binding molecules. The last column set in Fig. 10 reveals (i) disintegration of site I resulting in 92% release of bound WF, (ii) coordinative binding mode of the bpy complex which seems to be facilitated by denaturation of albumin. RhCp* itself displays stronger binding to unfolded HSA, that most probably can be explained by the appearance of more available coordination sites. The same experiment was performed for 12- and 25-fold excess of RuCym and RhCp* to the protein, respectively. After removal of non-bound metal species, SDS was added, and less than 1% of RhCp* and RuCym were liberated, which strongly suggests that all bound organometallic cations interact with HSA via coordinative bonds.

**Fig. 11:**
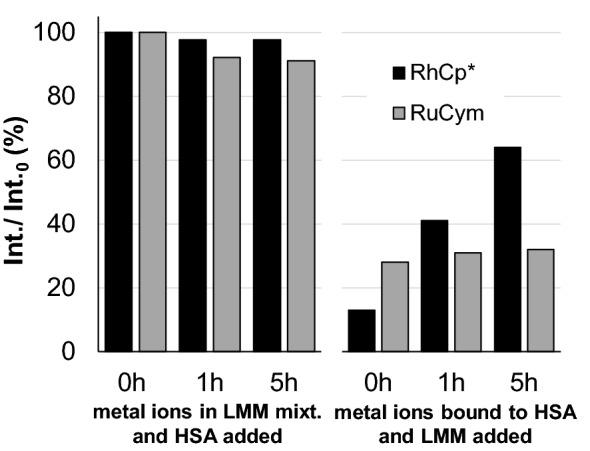
Effect of an LMM component mixture on the quenching efficacy of RhCp* (black) and RuCym (gray) at binding site I by addition of LMM compounds before (left side) or after (right side) equilibration of metallocompounds with HSA [*c*_HSA_ = 1 μM; *c*_Rh_ = *c*_Ru_ = 10 μM; *c*_LMM components_ = 77 μM (His), 23 μM (Met), 33 μM, (Cys), 100 μM (Ser), 99 μM (citrate), 1.5 mM (lactate); *λ*_EX_ = 295 nm, *λ*_EM_ = 350 nm; pre-equilibration = 2 h (Rh) or 30 h (Ru)]

The altered binding nature of the organometallic ions and their various complexes to HSA was proven in the previous sections, at the same time exploring the competitiveness of low-molecular-mass serum components is a further important task. The effect of single LMM serum compounds on the albumin binding of the metal ions was followed by fluorescence quenching experiments focusing on the binding event at site I. Human serum contains numerous possible interacting partners for organometallic complexes. We have chosen, by their relatively high serum concentrations, serum components Ser (~ 100 μM), His (~ 77 μM), Met (~ 77 μM) and Cys (~ 33 μM); citric acid (~ 99 μM) and sodium lactate (~ 1.5 mM). Figure 11a shows the quenching effect of RuCym and RhCp* in the presence of these LMM serum components at two different experimental sets: once organometallic cations were mixed with LMM components and HSA was added, or cations pre-equilibrated with HSA (bound to site I) were treated with LMM compounds. In the first experimental set, LMM mixture interfered strongly with the quenching effect, i.e., the interaction of these cations with site I. While site I-bound organometallic species in the second set showed no (RuCym) or rather slow (RhCp*, several hours) interaction with LMM compounds. Measured intensities for RhCp* tend to show similar values irrespective of the order of component addition (see Figs. 11 and S7a), namely binding towards LMM compounds is a thermodynamically favored, but rather slow process in case of the albumin-bound metal, pointing out the kinetic aspects of this interaction. The effectivity of each LMM compound against site I for RhCp* coordination was investigated as well and it follows the trend: His ~ Met ~ Cys > Ser > citrate ≥ lactate ~ buffer only. RuCym displays somewhat different preferences: citrate is as potent competitor as His and Cys; Met and Ser result in moderate quenching of fluorescence, while lactate has again no effect on binding at site I. The reaction is much slower for RuCym compared to RhCp* (see Fig. S7). Interestingly, addition of maltolato and dhp complexes of RuCym to the mixture of HSA and LMM compounds resulted in a fast drop of Trp fluorescence to 75% and 50%, respectively, that was followed by an increase in intensity taking several hours (Fig. S8). It seems that these ligands act as carriers enabling competition of LMM compounds for albumin-bound metal ions.

LMM components were applied here in their real serum concentration, but samples contained 1 μM HSA and tenfold excess of metallocompounds. Such a low concentration of HSA is due to the method requirements, but is much lower than the serum concentration. Taking into account this limitation, our main conclusions are as follows: (i) LMM serum components may be effective competitors of site I in binding of the two organometallic cations, (ii) on the other hand, protein-bound cations are hardly accessible for LMM competitors owing to kinetic reasons. Former conclusions most probably can be extended for further binding sites on HSA, since results of quenching experiments presented in former sections revealed both kinetic and thermodynamic similarities of site I to the average binding sites characterized in global binding studies. Applying strictly oxygen-free conditions, Cys showed itself to be a potent binder of both organometallic cations. The carrier-like effect of bidentate ligands maltol and dhp on the site I binding of RuCym requires more detailed investigations in the future.

## Conclusions

In this work, the binding interactions between half-sandwich organorhodium and organoruthenium compounds and HSA were investigated by means of separation techniques, ultrafiltration and CZE, and spectroscopic methods, UV–Vis spectrophotometry, spectrofluorometry and ^1^H NMR. Albumin binding of organometallic ions RhCp* and RuCym and their dhp and pic complexes with different thermodynamic stability and kinetic properties was investigated in first line. Kinetic aspects, binding strength and location, the nature and reversibility of these interactions were discussed. Low stability RuCym(maltolato), and complexes of picolinate derivatives 6-Mepic and 2-quinaldic acid formed with RhCp* were also included in our investigations to obtain more information on the relevant physico-chemical parameters having an effect on the nature and extent of binding.

Studies in RPMI cell culture medium pointed out the significant decomposition of the complexes RhCp*(dhp) and RhCp*(pic) due to the interaction with medium components (mainly amino acids). Consequently, cytotoxicity measured for lower stability complexes corresponds to the sum of efficacy measured for the metal ion and the ligand separately.

RuCym and RhCp* can bind to HSA in high extent (14 ± 1 and 24 ± 3 eq per protein, respectively) via coordinative interactions preferably with N-donor atoms of HSA. RhCp* interacts with HSA in few minutes, while binding of RuCym takes about 24 h. Binding of RuCym(dhp) and RuCym(maltolato) complexes however is much faster.

Metal complexes studied display different binding modes. Our results suggest the following mechanism for albumin binding: as initial step a protein donor atom coordinates monodentately to the metal center replacing the aqua (or chlorido) leaving group in the metal complex (associative binding), that is optionally followed by coordination of additional protein donor atom(s) and consecutive release of the original ligand (dissociative binding). The binding mechanism is governed by the thermodynamic stability and kinetic inertness of the complexes; lower stability and labile complexes (e.g. RhCp*(dhp)) bind in dissociative manner to HSA, while in the case of highly stable and more inert compound RuCym(pic) associative binding is predominant. Complexes RuCym(dhp) and RhCp*(pic) interact with HSA both in associative and dissociative manner.

All of the studied compounds interact at the hydrophobic binding sites I and II of HSA and most probably binding via complex dissociation is favored. Adduct formation at these sites may become relevant by interfering the blood transport of other molecules bound here.

The maximal bound number of metal complexes on HSA varies within 4 and 14, however at physiological concentrations, namely at high HSA excess, complete binding and formation of only 1:1 adducts is expected. HSA binding of RuCym, RhCp* and their low stability complexes is hindered at site I in the presence of LMM serum compounds being able to coordinate to the organometallic ions. The most relevant LMM competitors of HSA are His, Met and Cys for both metal ions. At the same time, HSA-bound cations are hardly accessible for LMM chelators owing to kinetic reasons. Denaturation of the protein does not lead to the release of the organometallic ions and RhCp*(bpy), on the contrary, bound fractions increased, because of the appearance of new accessible coordinating sites. This phenomenon raises the question whether HSA can deliver these organometallic compounds or serves as a less available pool for them. This is currently investigated in our laboratory. The great number of accessible binding sites providing coordinative interactions for the studied metal ions and their complexes is not a specific feature of albumin, most probably numerous different types of proteins can interact with these compounds in similar manner to HSA. In this way organorhodium and organoruthenium complexes being able to form coordinative bond(s) rapidly with proteins may lead to decrease of effective drug concentration and may be responsible for adverse effects as well.

## Electronic supplementary material

Below is the link to the electronic supplementary material.

Supplementary material 1 (PDF 1366 kb)

## Abbreviations

6-Mepic: 6-Methyl-2-picolinic acid
BGE: Background electrolyte
bpy: 2,2′-Bipyridine
CZE: Capillary zone electrophoresis
DG: Dansylglycine
dhp: 1,2-Dimethyl-3-hydroxy-pyridin-4(1H)-one, deferiprone
HMM: High molecular mass
HSA: Human serum albumin
LMM: Low molecular mass
maltol: 3-Hydroxy-2-methyl-pyran-4(1H)-one
PBS’: Modified phosphate buffered saline
pic: 2-Picolinic acid
PTA: 1,3,5-Triaza-7-phosphaadamantane
RhCp*: Rh(η^5^-C_5_Me_5_)
RM175: [Ru(II)(η^6^-biphenyl)(ethylenediamine)Cl]^+^
RuCym: Ru(η^6^-*p*-cymene)
UV–vis: UV–visible
WF: Warfarin
