# Supplementary material for: Binding mechanisms of half-sandwich Rh(III) and Ru(II) arene complexes on human serum albumin: a comparative study
Source: J Biol Inorg Chem. 2019 Jul 12;24(5):703–19. doi: 10.1007/s00775-019-01683-0 (PMC6682546; doi:10.1007/s00775-019-01683-0)
Supplement: Supplementary file 1 — Supplementary material 1 (PDF 1366 kb) [file 775_2019_1683_MOESM1_ESM.pdf]

SUPPLEMENTARY INFORMATION

Binding mechanisms of half-sandwich Rh(III) and Ru(II) arene complexes on human serum albumin: a comparative study

Orsolya Dömötör, Éva A. Enyedy

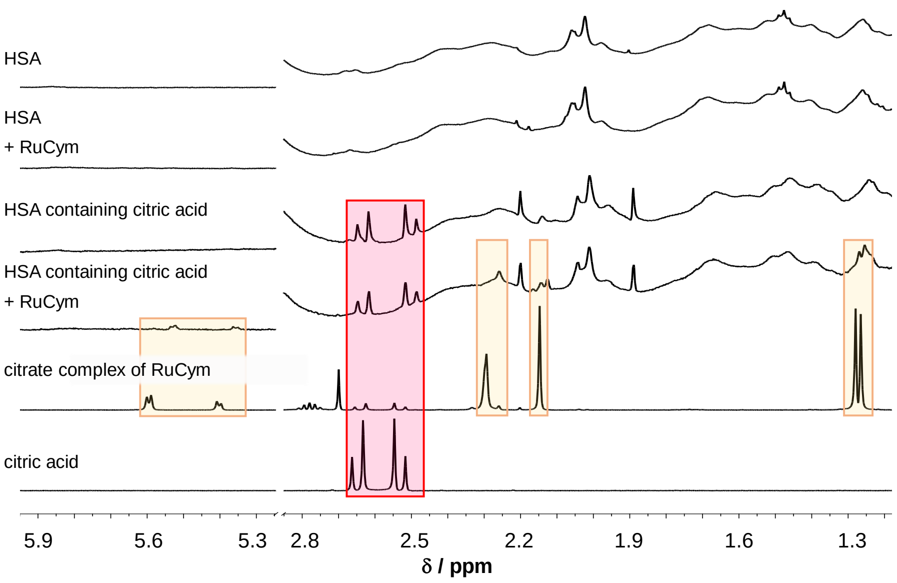

Fig. S1 ^{1}H NMR spectra of HSA and HSA – RuCym system in the absence and in the presence of citric acid contamination and the spectra of the citrate complex of RuCym and citric acid at pH = 7.40 {$c_{compound}$ ≈ 1 mM, c_{HSA} ≈ 0.5 mM}

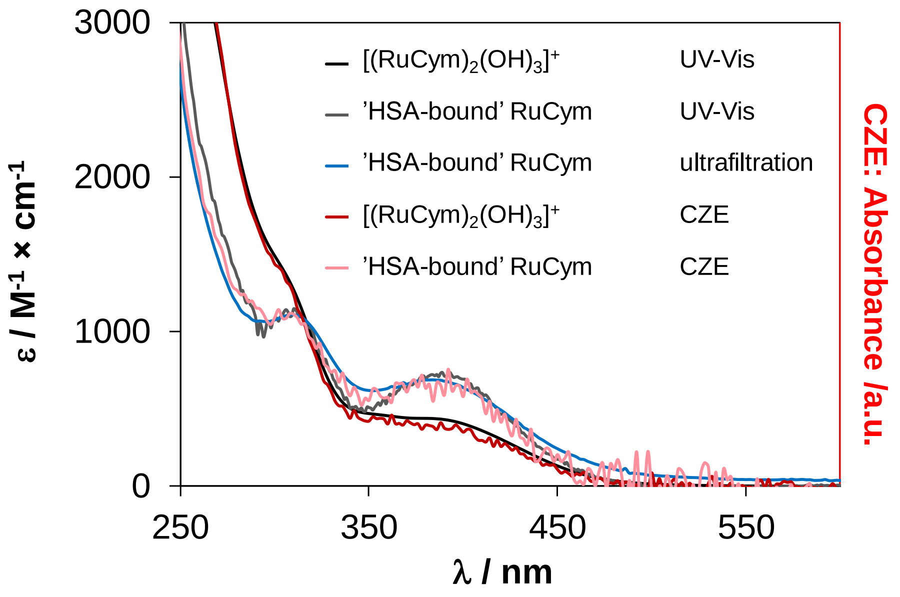

Fig. S2 UV–vis molar absorbance (ε) spectra (black grey and blue) derived from UV–vis steady-state and ultrafiltration measurements and normalized absorbance spectra (red and pink) obtained in CZE measurements for RuCym and its HSA-bound form; Spectra depicted here are subtracted by the corresponding absorbance spectrum of HSA {c_{HSA} = 30 μM (steady-state), 29 μM (ultrafiltration), 100 μM (CZE); HSA-to-metal ion ratios: 1:1 (steady-state), 1:10 (ultrafiltration), 1:5 (CZE); pH = 7.40 (PBS')}

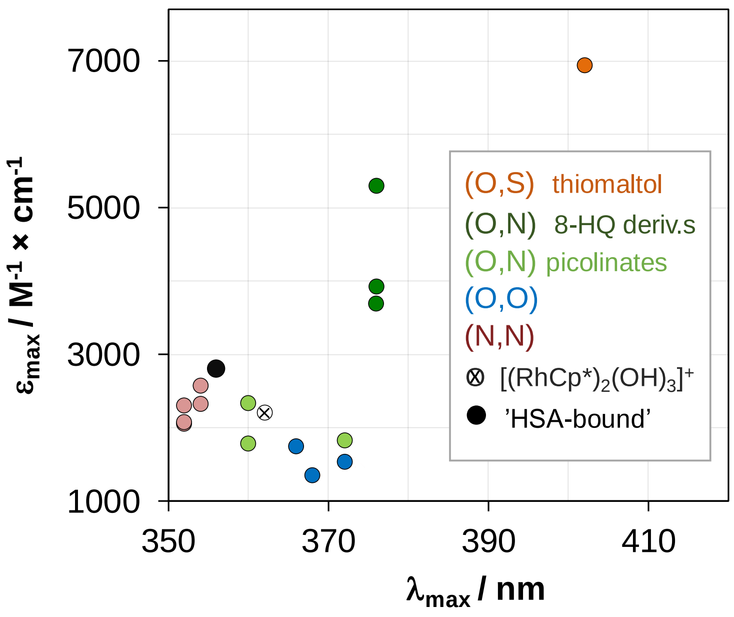

Fig. S3 Correlation diagram for the λ_{max} and ε_{max} values of various bidentate [RhCp*(L)(H_2O)] complexes and of dimeric hydroxide species $[(RhCp^*)_2(\mu\text{-}OH)_3]^+$ and HSA-bound RhCp* (see exact values for each complexes in Table S2).

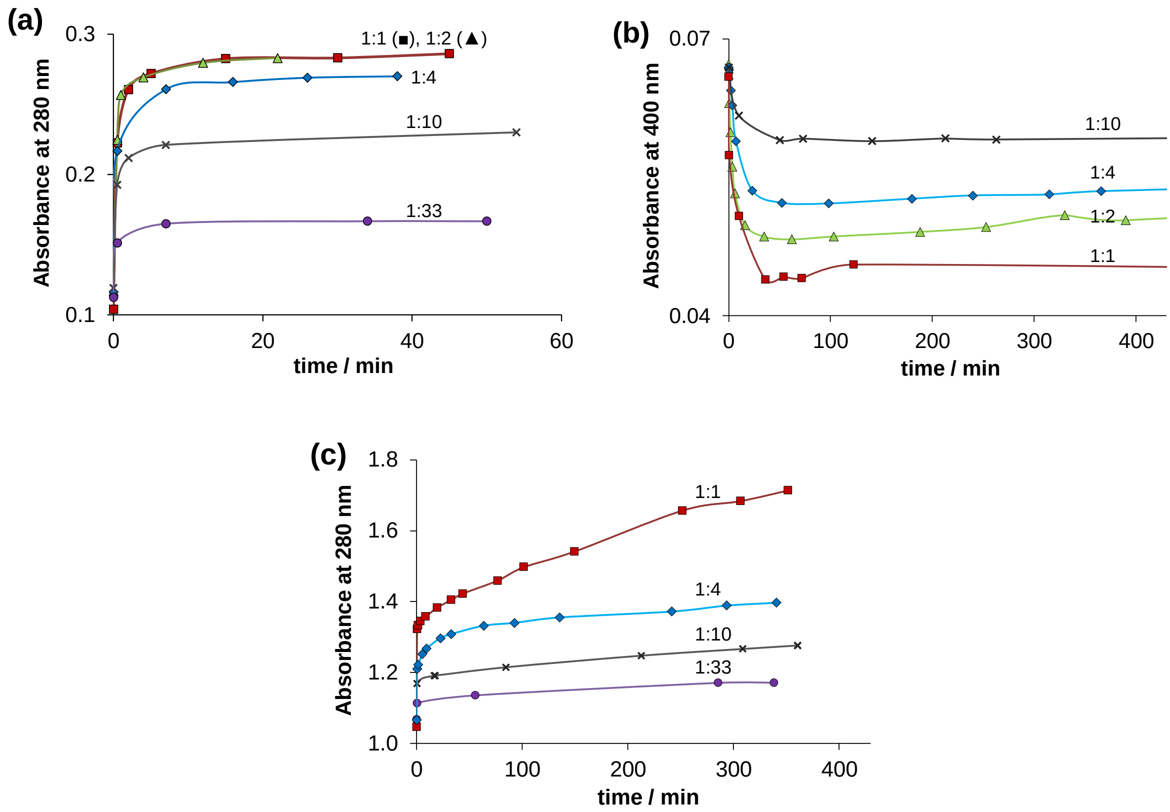

Fig. S4 UV–vis absorbance of RhCp* complexes of dhp (a) and pic (b) and absorbance of RuCym(dhp) (c) as a function of time in the presence of various complex-to-HSA ratios; Absorbances are subtracted by the corresponding absorbance of HSA, and were calculated for l = 1 cm {c_{comp} = 20 μM (a), 40 μM (b), 180 μM (c); PBS'; 25 °C}

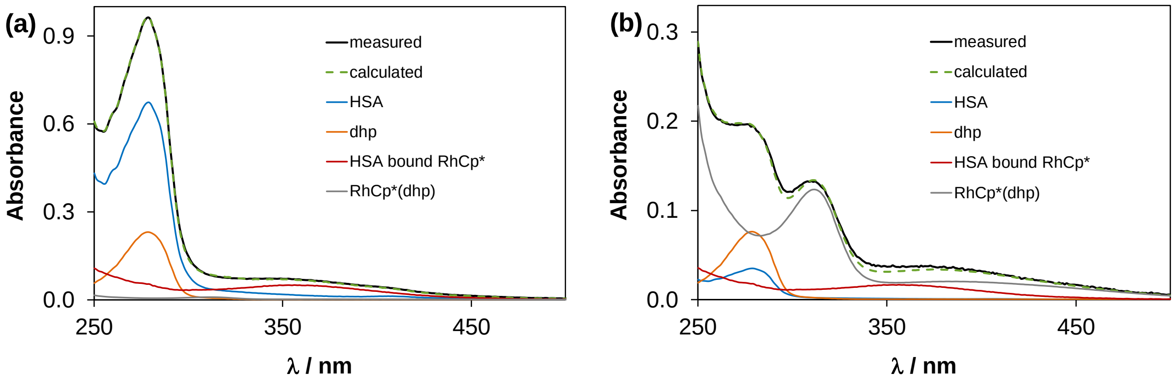

Fig. S5 Measured and fitted UV–vis spectra of HSA – RhCp*(dhp) 1:1 (a) and (1:20) (b) systems; Fitted spectrum is given as the sum of the spectra of the assumed species listed in the figure {$c_{complex}$ = 20 μM, PBS', 25 °C}

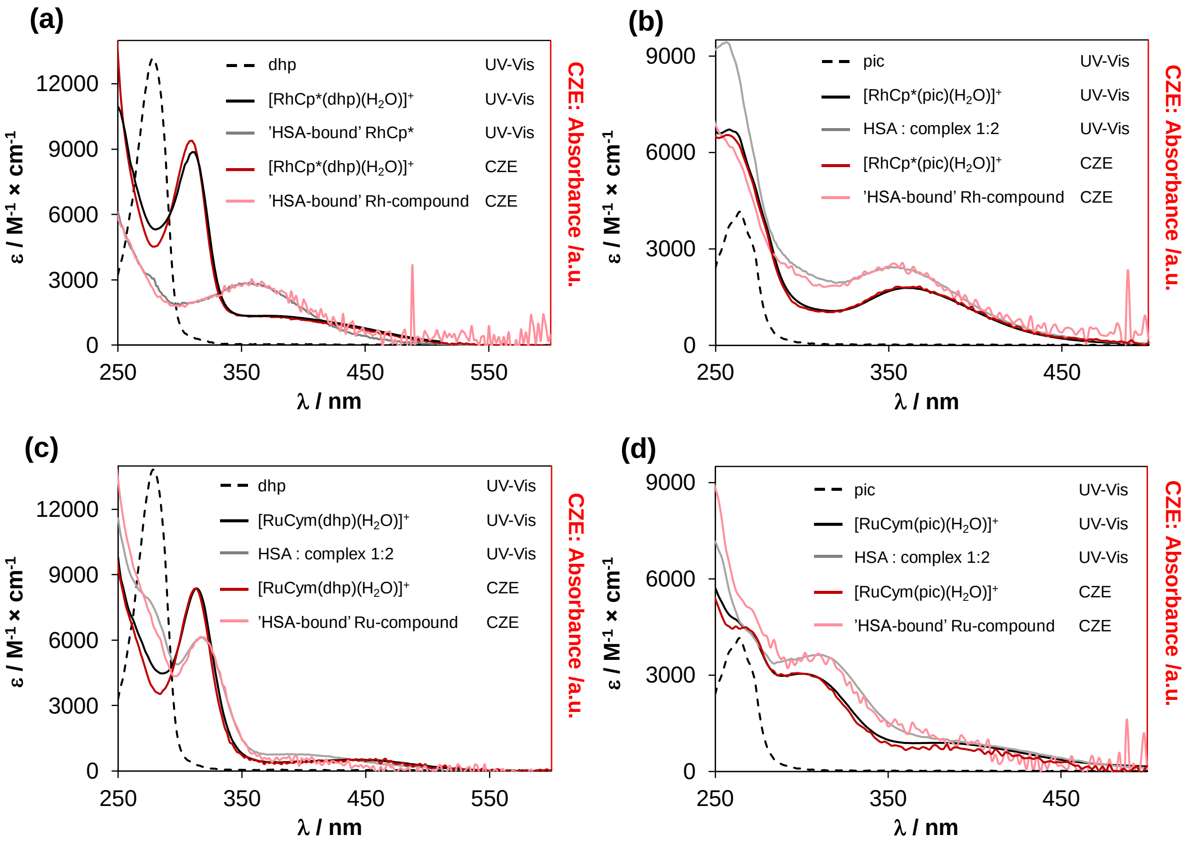

Fig. S6 UV–vis molar absorbance (ε) spectra (black and grey) and normalized absorbance spectra (red and pink) obtained in CZE measurements for the indicated compounds and compound−HSA systems; (a): $[RhCp^*(dhp)(H_2O)]^+$; (b): $[RhCp^*(pic)(H_2O)]^+$; (d): $[RuCym(dhp)(H_2O)]^+$; (d): $[RuCym(pic)(H_2O)]^+$; Spectra depicted here are subtracted by the corresponding absorbance spectrum of HSA {pH = 7.40 (PBS')}

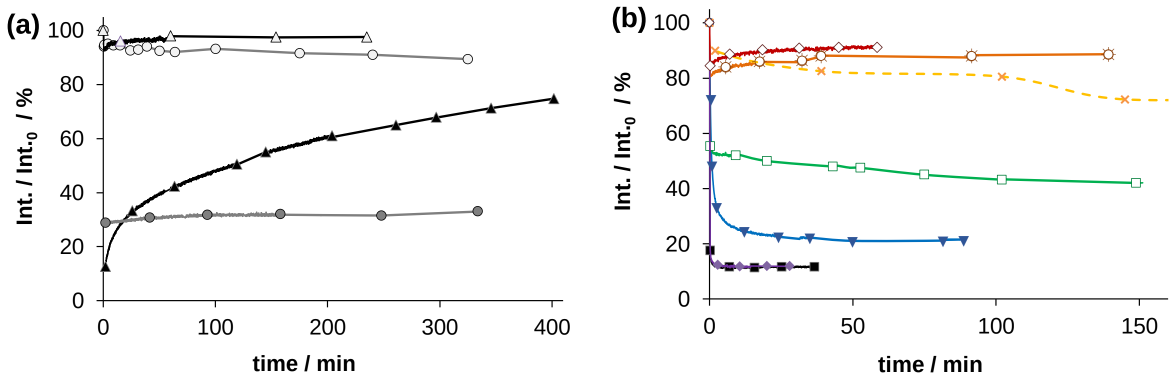

Fig. S7 Effect of an LMM component mixture on the quenching efficacy of RhCp* (▲,Δ) and RuCym (●,○) at binding site I by addition of LMM compounds before (empty symbols) and after (filled symbols) equilibration of metallocompounds with HSA (a). Effect of each

LMM component on the quenching effect of RhCp*: His (◊), Met (☼), Cys (×), Ser (□), citrate (▼) and lactate (♦); quenching curve without any LMM component recorded in buffer is shown as well (■) (b). {c_{HSA} = 1 µM; c_{Rh} = c_{Ru} = 10 µM; $c_{LMM\ components}$ = 77 µM (His), 23 µM (Met), 33 µM, (Cys), 100 µM (Ser), 99 µM (citrate), 1.5 mM (lactate); λ_{EX} = 295 nm, λ_{EM} = 350 nm; pre-equilibration = 2 h (Rh) or 30 h (Ru);}

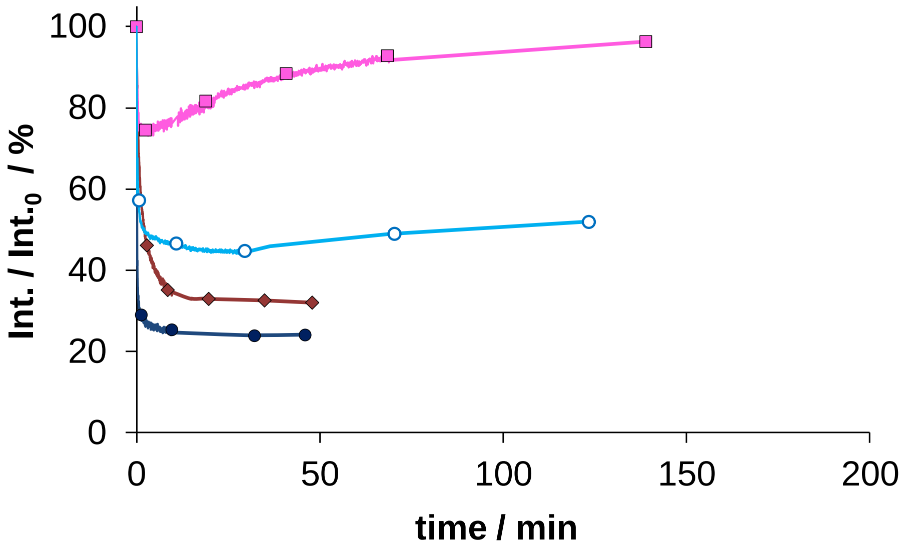

Fig. S8 Quenching effect of RuCym(maltolato) (■,♦) and RuCym(dhp) (○,●) at binding site I in the absence (♦,●) and in the presence (■,○) of LMM compounds {c_{HSA} = 1 µM; $c_{complex}$ = c_{Ru} = 10 µM; $c_{LMM\ components}$ = 77 µM (His), 23 µM (Met), 100 µM (Ser), 99 µM (citrate), 1.5 mM (lactate); λ_{EX} = 295 nm, λ_{EM} = 350 nm}

Table S1 Complex stability (%), chloride affinity (%) and *n*-octanol-water distribution coefficient ($D_{7.4}$) of the indicated RuCym and RhCp* species

	**stability**[a] complex (%)	**Cl^- affinity**[b] [M(L)(Cl)] (%)	**lipophilicity**[c] log $D_{7.40}$	**Reference**
**RuCym**	-	-	-0.46	[60]
complexed by				
maltol	66	-	-	[59]
dhp	96	-	-0.60(1)[a]	[59]
pic	99	71	-	[57,58]
**RhCp***	-	-	-0.61	[60]
complexed by				
dhp	93	38	-0.84(1)[a]	[50]
pic	99.5	94	-1.01	[56]
6-Mepic	99.5[d]	93	-0.84	[60]
2-quinaldic acid	99.3[d]	96	-0.02	[60]
ethylenediamine	>99.9	93	-	[49]
bpy	>99.9	97	-	[49]

[a] Calculated at 50 μM complex concentration, pH = 7.40, I = 0.2 M KCl
[b] c(complex) = 50 μM, I = 0.1 M KCl
[c] log *D* determined in *n*-octanol/water partition experiments at pH 7.40 (phosphate) containing 0.1 M KCl
[d] Determined at I = 0.2 M KNO_3

Table S2 λ_{max} and molar absorbance values (ε) of RhCp* residue (denoted as M) at different coordination environments {t = 25 °C}

**RhCp* at different conditions**		λ_{max} (nm) (ε_{max} ($M^{-1}\times cm^{-1}$))		Reference[a]
	HSA bound form at 1:1 ratio[b]	356 (2880)		-
	HSA bound form at 1:20 HSA-to-metal ion ratio[b]	373 (2270)		-
	$[RhCp^*(H_2O)_3]^{2+}$ [c]	374 (1540)		[50]
	$[(RhCp^*)_2(\mu\text{-}OH)_3]^{+}$ [c]	362 (2200 / 2)[d]		[50]
**bidentate ligand complexes[e]**		$[M(L)(H_2O)]$	$[M(L)(Cl)]$	
**(O,O)**	maltol	not measurable	396 (1680)	[50]
**(O,O)**	allomaltol	372 (1540)	398 (1750)	[50]
**(O,O)**	deferiprone	368 (1350)	404 (1450)	[56]
**(O,O)**	acetylacetone	366 (1750)	398 (1920)	[65]
**(O,S)**	thiomaltol	402 (6940)	418 (7480)	[74]
**(O,N)**	2-picolinic acid (pic)	360 (1790)	378 (1950)	[56]
**(O,N)**	6-Mepic	372 (1830)	382 (1980)	[60]
**(O,N)**	3-isoquinoline-2-carboxylic acid	360 (2340)	374 (2440)	[60]
**(O,N)**	8-hydroxyquinoline	376 (3920)	382 (4460)	[72]
**(O,N)**	8-hydroxyquinoline-5-sulfonic acid	376 (5300)	382 (5700)	[72]
**(O,N)**	7-(1-piperidinylmethyl)-8-hydroxyquinoline	376 (3690)	382 (4330)	[72]
**(N,N)**	1,10-phenantroline	354 (2570)	358 (2810)	[75]
**(N,N)**	2,2’-bipyridine (bpy)	352 (2060)	368 (2200)	[49]
**(N,N)**	2-picolylamine	352 (2080)	360 (2240)	[75]
**(N,N)**	ethylenediamine	354 (2330)	362 (2530)	[49]
**(N,N)**	N,N’-dimethylethylenediamine	352 (2310)	370 (2490)	[75]
**tris complex of monodentate ligand**		$[RhCp^*(L,L,L)]$		
**(N,N,N)**	3 × N-methylimidazole[f]	355 (2250)		-

[a] Experimental details can be found in the referred literature

[b] Measured in PBS’ (pH = 7.40)

[c] Derived from pH-dependent UV–vis titration in chloride-free medium (I = 0.2 M KNO_3)

[d] The dimeric hydroxido species containing two RhCp* moiety has a molar absorbance $\varepsilon = 1100\ M^{-1} \times cm^{-1}$

[e] Derived from H_2O/Cl^- exchange experiments followed by UV–vis

[f] Measured at pH 7.40 (PBS’) at $c_{Rh} = 150\ \mu M$ and $c_{MeIm} = 1.36$ mM
